# Vital Signs: Trends in Use of Long-Acting Reversible Contraception Among Teens Aged 15–19 Years Seeking Contraceptive Services — United States, 2005–2013

**Published:** 2015-04-10

**Authors:** Lisa Romero, Karen Pazol, Lee Warner, Lorrie Gavin, Susan Moskosky, Ghenet Besera, Ana Carolina Loyola Briceno, Tara Jatlaoui, Wanda Barfield

**Affiliations:** 1 Division of Reproductive Health, National Center for Chronic Disease Prevention and Health Promotion, CDC; 2 Office of Family Planning, Office of Population Affairs, US Department of Health and Human Services

## Abstract

**Background:**

Nationally, the use of long-acting reversible contraception (LARC), specifically intrauterine devices (IUDs) and implants, by teens remains low, despite their effectiveness, safety, and ease of use.

**Methods:**

To examine patterns in use of LARC among females aged 15–19 years seeking contraceptive services, CDC and the U.S. Department of Health and Human Services’ Office of Population Affairs analyzed 2005–2013 data from the Title X National Family Planning Program. Title X serves approximately 1 million teens each year and provides family planning and related preventive health services for low-income persons.

**Results:**

Use of LARC among teens* seeking contraceptive services at Title X service sites increased from 0.4% in 2005 to 7.1% in 2013 (p-value for trend <0.001). Of the 616,148 female teens seeking contraceptive services in 2013, 17,349 (2.8%) used IUDs, and 26,347 (4.3%) used implants. Use of LARC was higher among teens aged 18–19 years (7.6%) versus 15–17 years (6.5%) (p<0.001). The percentage of teens aged 15–19 years who used LARC varied widely by state, from 0.7% (Mississippi) to 25.8% (Colorado).

**Conclusions:**

Although use of LARC by teens remains low nationwide, efforts to improve access to LARC among teens seeking contraception at Title X service sites have increased use of these methods.

Implications for public health practice: Health centers that provide quality contraceptive services can facilitate use of LARC among teens seeking contraception. Strategies to address provider barriers to offering LARC include: 1) educating providers that LARC is safe for teens; 2) training providers on LARC insertion and a client-centered counseling approach that includes discussing the most effective contraceptive methods first; and 3) providing contraception at reduced or no cost to the client.

## Introduction

The teen birth rate in the United States has continued to decline during the past two decades, from 61.8 births per 1,000 teens aged 15–19 years in 1991 to an all-time low of 26.5 births per 1,000 teens in 2013 (1). Improved contraceptive use has contributed substantially to this decline (2); however, there were approximately 273,000 births to teens in 2013 (1), and the U.S. teen pregnancy rate remains up to seven times higher than in some developed countries (3). Teen childbearing has potential negative health, economic, and social consequences for mothers and their children (4), and each year costs the United States approximately $9.4 billion (5).

A key strategy for further reducing teen pregnancy is increasing awareness, access, and availability of long-acting reversible contraception (LARC), specifically intrauterine devices (IUDs) and implants. IUD use was more common among U.S. women in the 1970s before concerns about safety led to a decline; however, with approval of redesigned IUDs and implants, there has been growing interest in the use of LARC (6). LARC requires no effort after insertion, and can prevent unintended pregnancy for at least 3 to 10 years, depending on the type of LARC (7). During the first year of typical use, both IUDs and implants have lower failure rates (<1%) than oral contraceptives (9%) and condoms (18%) (8), the two methods teens use most often (9). Among teens, LARC also has high acceptability (10) and higher continuation rates than shorter-acting methods (11). Further, LARC is safe and appropriate for teens (12): major professional societies, including the American College of Obstetricians and Gynecologists and the American Academy of Pediatrics, have endorsed LARC as a first-line contraceptive choice for teens that can be combined with condoms to provide the best protection against pregnancy and sexually transmitted diseases (13,14).

***Key Points***

- Intrauterine devices (IUDs) and implants, known as Long-Acting Reversible Contraception (LARC), are the most effective types of birth control for teens. With use of LARC, less than 1% of users become pregnant during the first year of use.
- LARC is safe for teens, requires no effort after insertion, and can prevent pregnancy for 3 to 10 years.
- Nationally, use of LARC among teens has increased but still remains low (<5%).
- Strategies for removing barriers to LARC include: 1) educating providers that LARC is safe for teens, 2) training providers on LARC insertion and use of a client-centered counseling approach that includes discussing the most effective contraceptive methods first, and 3) providing contraception at reduced or no cost to the client.
- Efforts to address barriers at Title X service sites have increased the percentage of teens selecting LARC as their preferred contraceptive option from 0.4% in 2005 to 7.1% in 2013.
- Additional information is available at http://www.cdc.gov/vitalsigns.

National estimates suggest use of LARC among teens has increased but still remains low (<5%) (15,16). Common barriers to LARC use by teens include unfounded concerns about safety, high upfront costs, and lack of awareness about LARC (17,18). For example, in a nationally representative sample of U.S. publicly funded family planning clinics, LARC was discussed with teen clients at fewer than half of these clinics (18). Common challenges reported by clinic directors included cost (60%), staff concerns about IUD use among teens (47%), and lack of training on insertion of implants (47%) and IUDs (38%) (18).

The reported barriers to use of LARC prompted CDC and the U.S. Department of Health and Human Services’ Office of Population Affairs to analyze clinic data from the Title X National Family Planning Program. Since 1970, this program has provided cost-effective and confidential family planning and related preventive health services for low-income women and men; it serves approximately 1 million teens each year (19). The Title X National Family Planning Program encourages health care providers to offer LARC as an option for teens by increasing awareness of clinical guidelines on LARC for teens, training providers on LARC insertion and client-centered contraceptive counseling, and supporting community education and outreach. The Title X Program also helps its service sites to reduce financial barriers to LARC (e.g., by building capacity to bill third-party payers).

## Methods

To examine use of LARC among female teens aged 15–19 years attending service sites funded under the Title X National Family Planning Program, data from the 2005–2013 Family Planning Annual Report† were analyzed. These years include the period during which modern IUDs and implants were available for use by women of all ages, including teens. The Family Planning Annual Report contains data from all entities that receive Title X grants to support delivery of family planning and related preventive health services through approximately 4,200 service sites. This report includes data on the number and percentage of female family planning users aged 15–19 years by primary contraceptive method and age.

A family planning user was defined as a person who had at least one family planning encounter at a Title X service site in a calendar year. The primary contraceptive method was defined as the method adopted or continued at exit from the last encounter of that year. If a user reported more than one method, only the most effective method was recorded as the primary method. Female clients were excluded from analyses if they were pregnant or seeking pregnancy; they or their partner were sterile by means other than surgical sterilization; or they reported refraining from sexual intercourse. A small percentage of clients (range = 1.8%–5.3% by year) was excluded because the primary contraceptive method at their last encounter was unknown.

Reversible contraceptive methods were placed in three tiers based on the percentage of users who experience pregnancy during the first year of typical use: most effective (<1%), moderately effective (6%–12%), and least effective (≥18%) (8). The most effective methods included IUDs and implants; moderately effective methods included oral contraceptives, injectables (e.g., Depo-Provera), the contraceptive patch, the vaginal ring, and diaphragms; and least effective methods included condoms, contraceptive sponges, spermicides, fertility awareness-based methods, and other methods, including withdrawal. Trends over time and by age, region, and type of service site were evaluated using the Cochran-Mantel-Haenszel test statistic.

### Results

**FIGURE 1:**
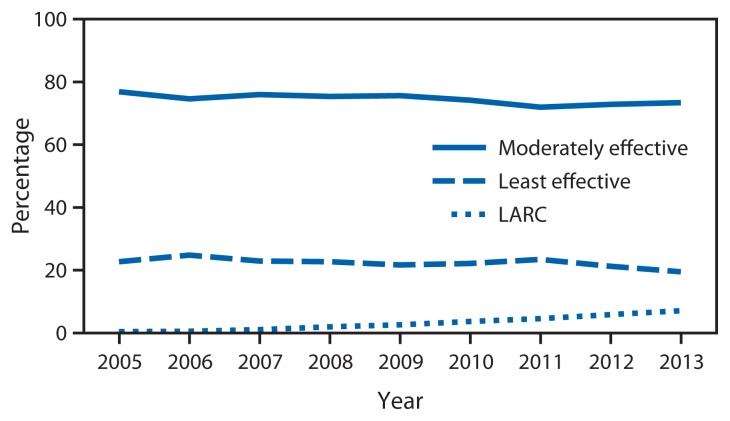
Percentage of female teens aged 15–19 years using moderately effective and least effective contraceptive methods, compared with long-acting reversible contraception (LARC), among those seeking contraceptive services at Title X service sites — United States, 2005–2013

Among approximately 7.5 million female clients aged 15–19 years who sought contraceptive services during 2005–2013 from Title X service sites in the United States, the percentage who adopted or continued use of LARC at their last visit increased from 0.4% (2005) to 7.1% (2013) (p-value for trend <0.001); the number of LARC users increased from 4,112 (2005) to 43,696 (2013). During this time, the percentage that used moderately effective methods decreased from 76.9% to 73.4%, and the percentage that used least effective methods decreased from 22.7% to 19.5% (Figure 1).

**FIGURE 2:**
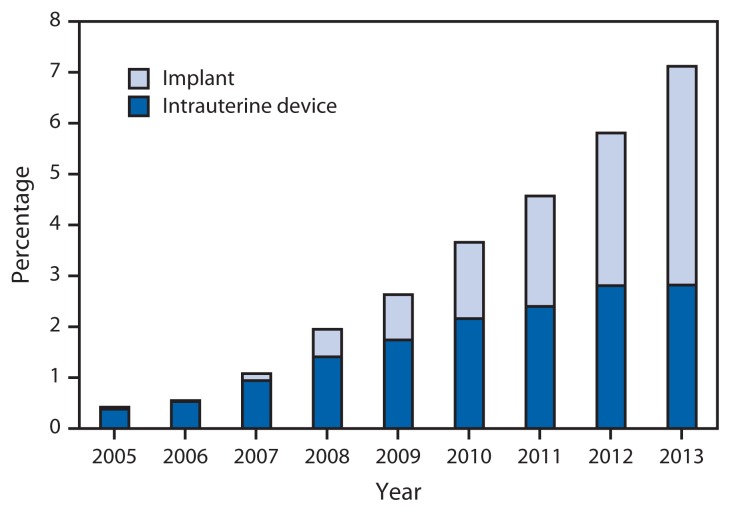
Percentage of female teens aged 15–19 years using long-acting reversible contraception (LARC) among those seeking contraceptive services at Title X service sites, by LARC type — United States, 2005–2013

By type of LARC, use of IUDs for teens aged 15–19 years increased from 3,685 (0.4%) to 17,349 (2.8%), and use of implants increased from 427 (0.04%) to 26,347 (4.3%) (Figure 2). Use of IUDs was more prevalent than use of implants during 2005–2011 but was surpassed by implants in 2012 and 2013.

By age, overall use of LARC during 2005–2013 was higher each year among teens aged 18–19 versus 15–17 years (p<0.001 for each year). Use of LARC increased from 0.6% to 7.6% among teens aged 18–19 years, and from 0.3% to 6.5% among teens aged 15–17 years. For both age groups, the increase in use of implants exceeded the increase in use of IUDs (teens 15–17 years: 0.05% to 4.5% for implants, and 0.2% to 2.0% for IUDs; teens 18–19 years: 0.04% to 4.1% for implants, and 0.5% to 3.4% for IUDs).

**FIGURE 3:**
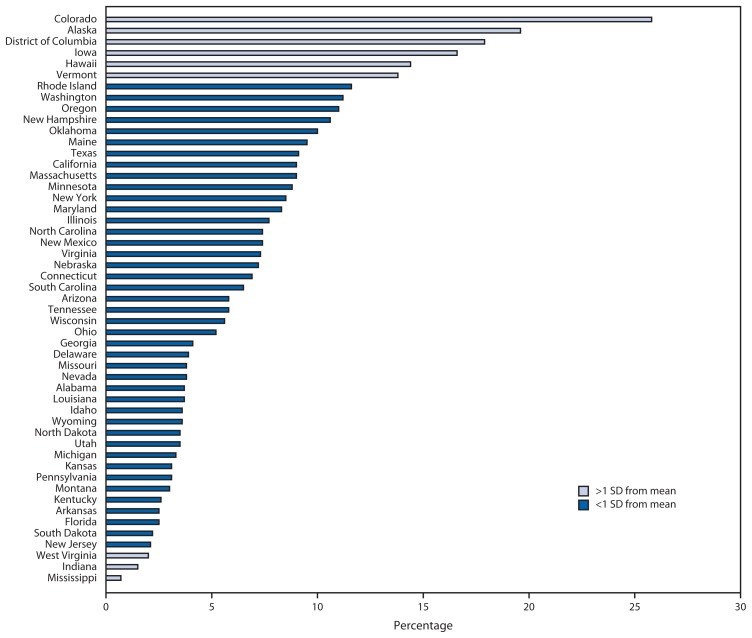
Percentage of female teens aged 15–19 years using long-acting reversible contraception (LARC) among those seeking contraceptive services at Title X service sites, by state — United States, 2013 **Abbreviation:** SD = standard deviation.

In 2013, among 616,148 female clients aged 15–19 years seeking contraception at Title X service sites, the use of LARC varied markedly by region (Table). Use was highest in the West (9.5%), followed by the Northeast and Midwest (both 6.4%), and lowest in the South (5.3%) (p<0.001). By state, Colorado had the highest percentage of teen clients using LARC (25.8%), followed by Alaska (19.6%), District of Columbia (17.9%), Iowa (16.6%), Hawaii (14.4%), and Vermont (13.8%); conversely, the lowest percentage of teen clients using LARC was in West Virginia (2.0%), Indiana (1.5%), and Mississippi (0.7%) (Figure 3). By type of LARC, use of IUDs was highest in Colorado (8.2%), Rhode Island (5.4%), New Hampshire (5.2%), and Washington (5.2%), and use of implants was highest in Colorado (17.6%), Alaska (15.4%), Iowa (13.4%), District of Columbia (12.9%), and Hawaii (12.2%) (Table).

Use of LARC among teens aged 15–19 years seeking contraception at Title X service sites also varied by type of facility. Service sites that focused primarily on delivering family planning services, as opposed to primary care services, had the highest percentage of teen clients using LARC (7.5%), followed by health departments (6.7%), other types of service sites (5.7%), and Federally Qualified Health Centers§ (5.6%) (p<0.001) (Table). By type of LARC, use of IUDs was highest at service sites that focused primarily on family planning services (3.3%), whereas use of implants was equally high (4.3%) at health departments and services sites that focused primarily on family planning services.

## Conclusions and Comment

These data show efforts to improve access to LARC among teens seeking contraception at Title X service sites have increased use of these methods more than 15-fold from 0.4% in 2005 to 7.1% in 2013, with a marked increase in use of implants. Concurrently, use of moderately effective and least effective methods among teens seeking contraceptive services declined. Given the estimated 4.4 million sexually experienced female teens in the United States (9), and the high effectiveness, safety and ease of using LARC, continued efforts are needed to increase access and availability of these methods for teens.

CDC, in partnership with the U.S. Department of Health and Human Services’ Office of Population Affairs, recently issued recommendations for providing quality family planning services, based on the Title X program’s guidance for direct service delivery (20). These recommendations outline a client-centered approach for contraceptive counseling, in which a client’s reproductive life plan, social needs, and contraceptive preferences are discussed along with medical information to identify acceptable methods for the client. By recommending that the most effective methods be discussed first, these recommendations promote increased awareness of LARC. In concurrence with statements from the American College of Obstetricians and Gynecologists and the American Academy of Pediatrics, these recommendations also emphasize the need to include information on the use of condoms for teens to reduce the risk for sexually transmitted diseases (13,14). Despite the long-term protection provided by LARC, it is important that teens have frequent follow-up to reinforce healthy decision-making, promote problem-solving regarding contraceptive continuation and sexually transmitted disease prevention, and receive other preventive health services (13).

Three other initiatives (21–23) have facilitated use of LARC among reproductive aged women, including teens, by underscoring the importance of educating providers that LARC is medically safe for teens (12), training providers on LARC insertion and use of a client-centered counseling approach that includes discussing the most effective contraceptive methods first (20), and providing contraception at reduced or no cost to the client. These efforts have increased the percentage of teens and young women selecting LARC as their preferred option for contraception and have been associated with declines in teen pregnancies, births, and abortions (21,22).

The findings of this report suggest that implants, as compared with IUDs, accounted for a greater proportion of the increase in use of LARC among teens seeking contraceptive services at Title X service sites. However, national surveys indicate that more service sites, whether privately or publicly funded, offer IUDs than implants on-site (24–26). To meet the increasing demand for implants by teens, providers should consider increasing on-site availability and affordability of implants.

This report documents that use of LARC among females aged 15–19 years seeking contraception through Title X was highest at services sites that focused primarily on delivering family planning services. This finding is consistent with a recent study of publicly funded clinics, in which those primarily focusing on family planning (compared with those focusing on primary care) offered more methods on-site, including IUDs and implants (24). Additionally, a 2011 survey of Federally Qualified Health Centers found that a higher percentage of centers receiving Title X funding (compared with those not receiving funding) offered IUDs and implants on-site (25). Together, these findings suggest the importance of providing quality contraceptive services, regardless of setting, to ensure that the contraceptive needs of teens are met.

The considerable state-specific variation observed in the prevalence of LARC use suggests that state-based policies and programs might also influence teen use of LARC. Over the past two decades, many states have expanded eligibility for Medicaid coverage of family planning services. Currently 25 states grant coverage solely on the basis of income, and in 20 states this expansion includes persons aged <19 years (27). Recent surveys have found that Title X service sites in states with Medicaid family planning expansions (compared with those without such expansions) are more likely to provide LARC on-site, report fewer cost-related difficulties obtaining LARC, have extended weekend and evening hours, have a higher percentage of clients paying for services with Medicaid, and assist clients with Medicaid enrollment (24).

The findings in this report are subject to at least three limitations. First, to minimize data collection burden for Title X grantees, only summary information on a limited number of client characteristics is requested for the Family Planning Annual Report. This limits the type of questions than can be addressed. For example, it is currently not possible to examine the use of the primary contraceptive method, including LARC, by factors such as race or ethnicity. Second, the use of existing clinic records might have been subject to error regarding the primary contraceptive method provided to teens; however, such records circumvent many of the biases associated with relying on self-report for sensitive behaviors. Finally, the Title X service sites provide care to those from underserved, primarily low-income communities nationwide, including teens, and might not be generalizable to the population of teens nationally. However, given the higher rates of unintended pregnancy among teens and low-income women (28), Title X data offer important information on a population with a high need for increased access to contraceptive services, including LARC.

This report documents increasing use of LARC among teens seeking contraceptive services at Title X service sites during the past decade. Approximately one out of every 14 teen clients seeking contraceptive services chose LARC as their preferred method. The type of data presented in this report can help identify areas where barriers remain and guide interventions to increase access to and awareness of LARC among teens. Removing barriers to LARC by educating providers that LARC is medically safe for teens, training providers on LARC insertion and a client-centered counseling approach that includes discussing the most effective contraceptive methods first, and providing contraception at reduced or no cost to the client, can increase the array of options available to teens and may contribute to the continuing declines in teen pregnancy in the United States.

## Figures and Tables

**TABLE:** Percentage of female Title X clients aged 15–19 years using long-acting reversible contraception (LARC), by age group, type of service site, region, and state — Family Planning Annual Report, United States, 2013

Characteristic	No.	% using LARC								

		15–19 yrs			15–17 yrs			18–19 yrs		
		
		Total	IUD	Implant	Total	IUD	Implant	Total	IUD	Implant
**Total**	**616,148**	**7.1**	**2.8**	**4.3**	**6.5**	**2.0**	**4.5**	**7.6**	**3.4**	**4.1**
**Type of service site**										
Health department	333,203	6.7	2.5	4.3	6.4	1.8	4.6	7.0	3.0	4.0
Family planning	277,000	7.5	3.3	4.3	6.6	2.2	4.4	8.2	4.0	4.2
FQHC	1,738	5.6	1.8	3.8	4.5	0.7	3.8	6.7	3.0	3.8
Other	4,207	5.7	1.9	3.9	4.8	0.8	4.0	6.4	2.7	3.8
**Region** *										
Northeast	115,850	6.4	3.2	3.2	5.8	2.4	3.4	6.9	3.9	3.1
Midwest	89,359	6.4	2.0	4.4	6.3	1.3	5.0	6.5	2.5	4.0
South	199,619	5.3	1.6	3.6	4.9	1.1	3.8	5.5	2.1	3.5
West	211,320	9.5	4.1	5.4	8.6	2.9	5.6	10.1	4.8	5.3
**State**										
Alabama	16,677	3.7	0.3	3.4	3.3	0.1	3.2	4.0	0.5	3.5
Alaska	1,207	19.6	4.1	15.4	18.6	2.9	15.8	20.3	5.1	15.1
Arizona	5,307	5.8	3.8	2.0	4.6	2.3	2.3	6.7	4.8	1.8
Arkansas	9,734	2.5	2.3	0.2	1.7	1.5	0.1	3.2	3.0	0.3
California	144,157	9.0	4.1	4.9	7.9	2.9	5.0	9.7	4.7	4.9
Colorado	9,211	25.8	8.2	17.6	24.8	6.3	18.6	26.6	9.8	16.8
Connecticut	5,556	6.9	2.4	4.4	6.4	1.7	4.8	7.2	3.0	4.2
Delaware	1,660	3.9	1.8	2.0	3.3	1.0	2.3	4.2	2.4	1.9
District of Columbia	2,116	17.9	5.0	12.9	14.9	2.7	12.2	20.3	6.9	13.4
Florida	22,027	2.5	2.0	0.5	1.8	1.3	0.6	3.1	2.6	0.5
Georgia	18,016	4.1	1.2	2.9	3.6	0.7	3.0	4.5	1.7	2.8
Hawaii	2,787	14.4	2.2	12.2	13.0	1.1	11.9	16.0	3.5	12.5
Idaho	3,539	3.6	2.9	0.7	1.9	1.5	0.4	5.3	4.3	0.9
Illinois	13,613	7.7	2.9	4.8	6.6	1.8	4.9	8.4	3.8	4.7
Indiana	4,539	1.5	0.7	0.9	1.1	0.6	0.5	1.8	0.7	1.1
Iowa	9,402	16.6	3.2	13.4	17.7	2.2	15.5	15.7	4.0	11.7
Kansas	3,890	3.1	1.8	1.3	2.8	1.5	1.4	3.3	2.0	1.3
Kentucky	8,787	2.6	0.5	2.1	2.9	0.1	2.8	2.4	0.7	1.7
Louisiana	5,708	3.7	0.6	3.1	3.6	0.2	3.5	3.7	0.9	2.9
Maine	3,673	9.5	4.6	4.8	9.0	3.3	5.7	9.9	5.9	4.0
Maryland	8,436	8.3	3.3	5.0	7.5	2.1	5.5	9.0	4.4	4.6
Massachusetts	8,905	9.0	3.5	5.4	7.0	2.1	4.9	10.7	4.8	5.9
Michigan	15,165	3.3	1.2	2.1	3.2	0.9	2.4	3.4	1.5	1.9
Minnesota	8,258	8.8	2.5	6.3	9.5	1.4	8.2	8.4	3.1	5.3
Mississippi	12,089	0.7	0.5	0.2	0.4	0.3	0.1	0.9	0.7	0.3
Missouri	9,146	3.8	0.9	2.9	4.2	0.7	3.5	3.4	1.1	2.2
Montana	4,382	3.0	1.5	1.5	2.7	1.0	1.7	3.2	1.9	1.2
Nebraska	2,887	7.2	3.1	4.1	6.2	2.0	4.2	7.8	3.8	4.0
Nevada	2,747	3.8	2.1	1.7	2.4	1.2	1.3	5.0	2.9	2.0
New Hampshire	2,982	10.6	5.2	5.4	10.1	3.7	6.4	11.0	6.4	4.6
New Jersey	10,519	2.1	1.6	0.5	1.4	1.0	0.5	2.5	2.0	0.5
New Mexico	5,064	7.4	2.2	5.3	5.0	1.2	3.8	9.5	3.0	6.5
New York	43,748	8.5	4.8	3.7	8.0	3.8	4.1	8.9	5.5	3.4
North Carolina	16,584	7.4	2.8	4.6	7.0	1.8	5.2	7.7	3.5	4.2
North Dakota	1,661	3.5	1.2	2.3	4.4	0.9	3.4	2.9	1.4	1.6
Ohio	12,599	5.2	1.7	3.5	5.3	1.2	4.1	5.2	2.2	3.0
Oklahoma	10,438	10.0	1.4	8.6	10.1	0.9	9.1	10.0	1.9	8.1
Oregon	9,949	11.0	4.5	6.5	10.4	3.3	7.1	11.5	5.7	5.8
Pennsylvania	36,229	3.1	1.2	1.9	2.8	1.0	1.8	3.4	1.4	2.0
Rhode Island	2,706	11.6	5.4	6.2	10.8	3.3	7.5	12.2	6.9	5.4
South Carolina	10,316	6.5	1.8	4.7	6.8	1.5	5.3	6.4	1.9	4.5
South Dakota	1,564	2.2	1.5	0.8	1.6	0.9	0.7	2.6	1.8	0.8
Tennessee	17,370	5.8	1.2	4.5	6.2	0.7	5.5	5.4	1.6	3.8
Texas	18,583	9.1	2.6	6.5	8.2	1.8	6.4	9.7	3.2	6.5
Utah	6,679	3.5	2.5	1.0	2.8	1.6	1.2	3.9	3.0	0.9
Vermont	1,532	13.8	4.2	9.5	13.4	2.3	11.1	14.1	5.9	8.3
Virginia	11,620	7.3	1.7	5.6	7.7	1.9	5.8	7.1	1.6	5.5
Washington	14,457	11.2	5.2	6.1	10.6	4.2	6.4	11.7	5.9	5.8
West Virginia	9,458	2.0	1.0	1.0	1.8	0.7	1.1	2.2	1.3	0.9
Wisconsin	6,635	5.6	2.0	3.6	4.7	0.9	3.8	6.1	2.6	3.5
Wyoming	1,834	3.6	0.8	2.8	3.0	0.4	2.6	4.1	1.2	2.9

**Abbreviations:** IUD = intrauterine device; FQHC = federally qualified health center.

* *Northeast:* Connecticut, Maine, Massachusetts, New Hampshire, New Jersey, New York, Pennsylvania, Rhode Island, Vermont. *Midwest:* Illinois, Iowa, Indiana, Kansas, Michigan, Minnesota, Missouri, Nebraska, North Dakota, Ohio, South Dakota, Wisconsin. *South:* Alabama, Arkansas, Delaware, District of Columbia, Florida, Georgia, Kentucky, Louisiana, Maryland, Mississippi, North Carolina, Oklahoma, South Carolina, Texas, Tennessee, Virginia, West Virginia. *West:* Alaska, Arizona, California, Colorado, Hawaii, Idaho, Montana, Nevada, New Mexico, Oregon, Utah, Washington, Wyoming.

## Footnotes

† Available at http://www.hhs.gov/opa/title-x-family-planning/research-and-data/fp-annual-reports.

§ Federally Qualified Health Centers are “safety net” providers such as community health centers, public housing centers, outpatient health programs funded by the Indian Health Service, and programs serving migrants and the homeless. The main purpose of these centers is to enhance the provision of primary care services in underserved urban and rural communities.
